# Increase in Muscle Mitochondrial Biogenesis Does Not Prevent Muscle Loss but Increased Tumor Size in a Mouse Model of Acute Cancer-Induced Cachexia

**DOI:** 10.1371/journal.pone.0033426

**Published:** 2012-03-12

**Authors:** Xiao Wang, Alicia M. Pickrell, Teresa A. Zimmers, Carlos T. Moraes

**Affiliations:** 1 The Sheila and David Fuente Graduate Program in Cancer Biology, University of Miami Miller School of Medicine, Miami, Florida, United States of America; 2 Neuroscience Graduate Program, University of Miami Miller School of Medicine, Miami, Florida, United States of America; 3 Sylvester Comprehensive Cancer Center, University of Miami Miller School of Medicine, Miami, Florida, United States of America; 4 Department of Neurology, University of Miami Miller School of Medicine, Miami, Florida, United States of America; Hospital Vall d'Hebron, Spain

## Abstract

Cancer-associated cachexia is a complex metabolic condition characterized by the progressive loss of body fat and deterioration of muscle mass. Although the cellular and molecular mechanisms of cachexia are incompletely understood, previous studies have suggested mitochondrial dysfunction in murine models of cancer cachexia. To better understand the metabolic shift in cancer-induced cachexia, we studied the effects of enhanced oxidative capacity on muscle wasting using transgenic mice over-expressing Peroxisome Proliferator-Activated Receptor gamma Co-activator-1α (PGC-1α) in skeletal muscle in a Lewis lung carcinoma-implanted model. Increased mitochondrial biogenesis was observed in the skeletal muscle of tumor-implanted mice. However, these increases did not prevent or reverse muscle wasting in mice harboring tumors. Moreover, tumor size was increased in muscle PGC-1α over-expressing mice. We found similar levels of circulating inflammatory cytokines in tumor-implanted animals, which was not affected by increased muscle expression of PGC-1α. Our data indicated that increased mitochondrial biogenesis in skeletal muscle is not sufficient to rescue tumor-associated, acute muscle loss, and could promote tumor growth, possibly through the release of myokines.

## Introduction

Clinically, cachexia is defined as “a complex metabolic syndrome associated with underlying illness and characterized by loss of muscle with or without loss of fat mass” [1]. It has been found in many chronic or end-stage diseases such as AIDS, tuberculosis, and cancer [2]. Up to 50% of untreated cancer patients experience progressive loss of fat and lean body mass without starvation, a complex syndrome referred to as cancer-induced cachexia [3]. The presence of wasting is usually associated with intolerance to treatment, poor quality of life and high mortality in patients [4].

Although extensive studies have been carried out during the last decade, the underlying mechanisms causing cancer cachexia are still not fully understood. One of the leading theories is that tumor-derived factors are responsible for the degradation of body mass, including the muscle [2]. It is widely accepted that pro-inflammatory cytokines play a key role in all pathways that lead to hyper catabolism and weight loss associated with cancer cachexia [5]. The presence of systemic inflammation is usually linked to worse prognosis in the patients [6].

Cancer cachexia causes systemic changes in patients' metabolic profile in order to support tumor development. It has been reported that mitochondrial dysfunction in the skeletal muscle, including decreased oxidative phosphorylation (OXPHOS) capacity and disrupted mitochondrial dynamics, is involved with systemic inflammation and skeletal muscle wasting [7]. The peroxisome proliferator-activated receptors (PPARs) transcription factors family and their modulator PPAR-gamma co-activator-1α (PGC-1α) are the master regulators of mitochondrial biogenesis and energy metabolism [8]. Mitochondrial uncoupling proteins (UCPs) 1, 2, and 3 are upregulated in atrophying muscle; and metabolic abnormality with increased proteolysis in the muscle has been implied in cachectic patients [9], [10]. The activation of pro-inflammatory cytokine TNFα-induced NF-κB was shown to decrease promoter transactivation and transcriptional activity of regulators of mitochondrial biogenesis (PGC-1α, PPARα, and TFAM) and affect downstream oxidative markers (citrate synthase, and cytochrome *c* oxidase) [11].

Clinical interventions have been developed for general symptom management of this devastating condition; however, these measures are only palliative without specifically targeting the causing factor of cachexia and the outcomes are not satisfactory [12].

In this study, we investigated the potential therapeutic effect of increasing mitochondrial biogenesis by overexpressing PGC-1α in the skeletal muscle in a transgenic mouse model of cancer cachexia. Our results indicate that increased mitochondrial biogenesis in the muscle was not sufficient to alter the levels of proinflammatory cytokines and prevent the muscle loss associated with tumor implantation. Moreover, the increase in muscle PGC-1α may also have the side effect of promoting tumor growth.

## Results

### Tumor-inoculated transgenic MCK-PGC-1α mice maintain increased mitochondrial biogenesis in gastrocnemius and quadriceps

**Figure 1:**
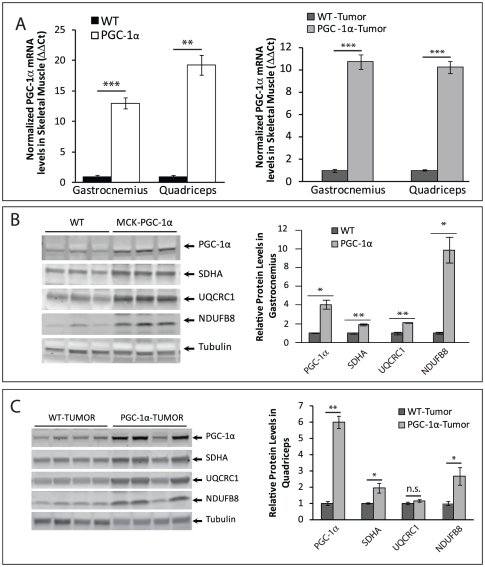
Tumor inoculated transgenic MCK-PGC-1α mice maintain upregulation of PGC-1α and mitochondrial markers. ***A*** **.** mRNA levels (ΔΔCt) of *Ppargc1a* in gastrocnemius and quadriceps normalized to *Gapdh* at 4 months-of-age in MCK-PGC-1α and controls without and with tumor implantation (n = 4/group). ***B, C*** **.** Left panel: Representative Western blotting analysis of steady state levels of PGC-1α and mitochondrial proteins (complex II subunit SDHA, complex III subunit UQCRC1, and complex I subunit NDUFB8) at 4 months-of-age from muscle homogenates of MCK-PGC-1α and controls. Right panel: Optical density (O.D.) quantification of proteins of interest normalized toα tubulin (n = 3/group in *B*, n = 4/group in *C*). Error bars are mean ± SEM.

Transgenic MCK-PGC-1α mice over-express PGC-1α in the skeletal muscle, driven by the muscle creatine kinase (MCK) promoter [13]. We observed an increase of *Ppargc1a* mRNA levels of 13-fold in the gastrocnemius, a muscle composed of similar levels of Type I (oxidative) and Type II (glycolytic) fibers and 19-fold in the quadriceps, a muscle composed mostly of Type II fibers, in 4-month-old tumor-free transgenic MCK-PGC-1α mice (Figure 1A). A similar increase was observed for tumor bearing mice (Figure 1A). When we determined the steady state levels of PGC-1α protein in gastrocnemius and quadriceps homogenates, we observed a marked increase in transgenic mice compared to controls, with or without tumors. The results for gastrocnemious and quadriceps were essentially identical for both genotypes (Figure 1B, C and not shown).

**Figure 2:**
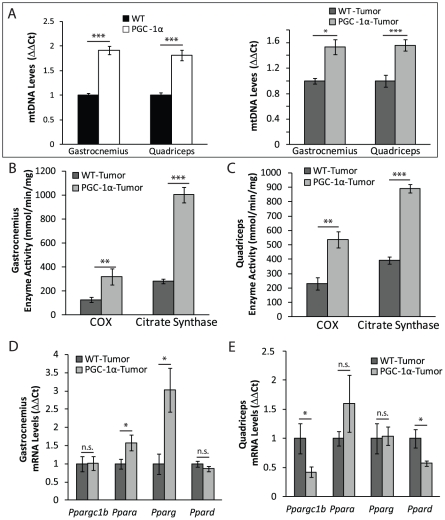
Tumor injected transgenic MCK-PGC-1α mice maintain upregulation of mtDNA levels and mitochondrial enzymes activity. ***A.*** mtDNA copy number of gastrocnemius and quadriceps of 4-months-old MCK-PGC-1α and wild-type mice, without and with tumor implantation (n = 5/group). *B–C.* COX and CS enzymatic activity of gastrocnemius (***B***) and quadriceps (***C***) muscle homogenates normalized to protein from tumor MCK-PGC-1α and age-matched controls (n = 5/group). *D–E.* mRNA levels (ΔΔCt) of *Ppara*, *Pparg*, *Ppard*, and *Ppargc1b* in gastrocnemius (*D*) and quadriceps (*E*) normalized to *Gapdh* at 4-months-old tumor MCK-PGC1–α and aged-matched controls (n = 4/group). Error bars are mean ± SEM.

PGC-1α is a transcriptional coactivator that upregulates the transcription of nuclear-coded mitochondrial proteins, stimulating mitochondrial biogenesis [14], [15], [16]. We quantified the levels of mitochondrial proteins in 4-month-old MCK-PGC-1αmice and found significantly higher levels of several mitochondrial markers in mice without or with tumors (Figure 1B and 1C). The levels of mtDNA in both gastrocnemius and quadriceps were elevated in PGC-1α expressing mice (Figure 2A). In concordance with signs of increased mitochondrial biogenesis, we observed significant increases in citrate synthase (CS) and cytochrome *c* oxidase (COX) activities both in gastrocnemius and quadriceps (Figure 2B, C).

Interestingly, we noticed a differential regulation of another member of the PGC-1 family, PGC-1β in gastrocnemius and quadriceps in tumor inoculated animals. While it remained unchanged in the gastrocnemius tissue, *Ppargc1b* mRNA levels were significantly decreased in quadriceps tissue of tumor-bearing MCK-PGC-1αmice (Figure 2D, E). Accordingly, the expression levels of transcription factors *Ppara* and *Pparg* were significantly increased in gastrocnemius but not in quadriceps muscle (Figure 2D, E).

### Over-expression of MCK-PGC-1αdoes not protect against cancer-induced muscle loss

Previously, our laboratory showed that over-expression of PGC-1αin skeletal muscle protected and slowed down the progression of mitochondrial myopathies and age-induced sarcopenia [17], [18]. We hypothesized that with increased mitochondrial function, our MCK-PGC-1α mice would be more resistant to muscle wasting by reversing the metabolic changes contributing to cachexia.

**Figure 3:**
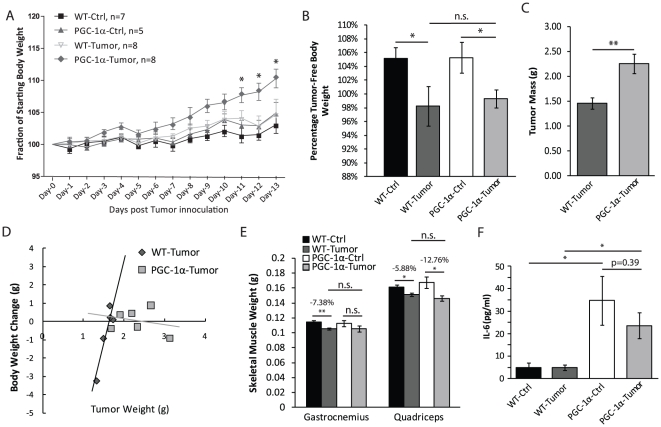
Over-expression of MCK-PGC–1α does not protect against cancer-induced cachexia. ***A.*** Percentage body weight over two weeks after tumor cell or saline injection for MCK-PGC-1α or controls at 4 months-of-age, numbers of animals as labeled. ***B.*** Percentage tumor-free body weight at 2 weeks after tumor inoculation of MCK-PGC-1α and age-matched controls, numbers of animals as in panel ***A***. ***C.*** Weight of tumor (grams) extracted from site of injection 2 weeks after tumor inoculation (n = 5 for wild-type, n = 7 for MCK-PGC-1α). ***D.*** Linear regression modeling relationship between changes in percentage body weight and the weight of tumor of MCK-PGC-1α and age-matched tumor-bearing controls, numbers of animals as in panel ***C***. ***E.*** Weight of gastrocnemius and quadriceps (grams) of saline-injected and tumor-inoculated groups of MCK-PGC-1α or age-matched wild-type mice, numbers of animals as in panel ***A***. ***F.*** Concentrations of serum IL-6 in control and tumor-bearing wild-type and MCK-PGC-1α mice (n = 5/group). Error bars are mean ± SEM.

After confirming this robust enhancement in mitochondrial biogenesis, we examined whether over-expression of PGC-1α could provide protection against muscle loss in our tumorigenic model. We followed the weight of the MCK-PGC-1α and wild-type mice, with or without tumor, as an indicator of general health after inoculating tumor cells. We observed no significant difference between groups until post-injection day-12 and -13, where MCK-PGC-1α tumor mice had a significant increase in percentage body weight as compared to all 3 other groups of mice (Figure 3A).

To understand where this increase in body weight for the MCK-PGC-1α tumor mice came from, we examined the body weight and extracted tumor weight. We found that tumor-free body weight was significantly decreased in tumor-bearing mice of both genotypes, and no major differences between the transgenic and wild-type mice (Figure 3B). However, the tumors extracted from MCK- PGC-1α mice were approximately 50% larger than controls (Figure 3C). When plotting the changes of body weight against tumor size, we noted a positive correlation in wild-type mice, which was disrupted in MCK-PGC-1α tumor mice (Figure 3D). With this evidence, we concluded that the total body weight gain for tumor-bearing MCK-PGC-1α mice was caused by the increase in tumor mass and not a gain in muscle weight. However, despite having larger tumors, MCK-PGC-1α mice did not lose more weight than tumor bearing controls.

The weights of both gastrocnemius and quadriceps were mildly, but significantly decreased in wild-type tumor mice, indicating our model induced muscle loss (Figure 3E). However, there was no difference in either muscle group as compared between tumor-inoculated transgenic and wild-type mice (Figure 3E). We also quantified the concentration of pro-inflammatory cytokine IL-6 in the serum. As expected, IL-6 levels were dramatically increased in tumor-bearing mice of both genotypes compared to tumor-free mice, but we did not find significant differences between the two genotypes (Figure 3F). Thus, MCK-PGC-1α did not seem to protect against muscle loss or to lower systemic IL-6 levels at this endpoint.

## Discussion

In this study, we utilized MCK-PGC-1α mouse model to study the effect of increased mitochondrial biogenesis on cancer-induced muscle loss. We found increased mitochondrial biogenesis in the muscle of MCK-PGC-1α tumor mice compared to WT tumor mice. Surprisingly, we found that the increased expression of PGC-1α in muscle did not prevent muscle loss. This was an unexpected result, as muscle PGC-1α was shown by our group and others to confer broad protection to different conditions associated with muscle degeneration [17], [19].

Although the causes of cachexia are still poorly understood, increased degradation and decreased synthesis of muscle proteins by the proteasome system appears to play a major role [20]. Systemic inflammation seems to mediate this mechanism in cancer-induced cachexia [5]. Rosenberg and colleagues proposed that high serum levels of TNFα and IL-1β were the causes for weight loss in rheumatoid cachexia [21]. Systemic cytokine-driven inflammatory response in AIDS patients with concurrent active infections has also been associated with cachexia [22]. The model of subcutaneous tumor implantation also shows an increase in circulating IL-6 levels associated with muscle and total body weight loss. However, in our experiments the IL-6 levels were not different between wild-type and MCK-PGC-1α mice (Figure 3F). Therefore, we speculate that although PGC-1α can protect against muscle loss associated with intrinsic metabolic dysfunctions [17], [19], it is less effective in precluding muscle loss caused by extrinsic inflammatory signals.

The MCK-PGC-1α mouse model has been extensively studied by our group and others in various myopathies. Besides the transgenic strategy, PGC-1α can also be induced by endurance exercise [23], [24]. Both increased muscle PGC-1α expression and exercise have been shown to ameliorate systemic inflammation [9], [19], [25]. As part of combinatory therapy, exercise concordant with patient's physical conditions is usually suggested during clinical intervention [26]. In our model of cancer-induced cachexia experimental animals die within 2 weeks after tumor inoculation. The mice developed mild muscle loss within a 13-day time frame with the tumor appearing as early as day 5 after inoculation. Although PGC-1α overexpression did not ameliorate the mild muscle loss in our acute tumorigenic model, we believe future experiments should examine the potential therapeutic effect of increased mitochondrial biogenesis in more severe or chronic models of cancer-induced muscle wasting.

Although the reason for this negative result is not known, we also found that the tumors were approximately 50% larger in mice overexpressing muscle PGC-1α. One possible explanation for this observation is that skeletal muscle can secrete myokines, such as IL-6, which could have growth promoting activity [27]. This was an expected observation that warrants further exploration with different tumor types and models.

In summary, our work demonstrated that stimulating mitochondrial biogenesis was not sufficient to prevent or reverse muscle loss during acute cancer-induced muscle wasting. Moreover, we found evidence that PGC-1α expression in muscle can lead to the development of larger tumors.

## Methods

### Animals

The generation of MCK-PGC-1α transgenic mice was previously described [13]. Female animals for analysis were pure C57BL/6J MCK-PGC-1α mice with age-matched littermate controls. All mice procedures were performed according to a protocol approved by the University of Miami: Institutional Animal Care and Use Committee (#10-071). Mice were housed in a virus-antigen-free facility at the University of Miami: Division of Veterinary Resources under a 12 hr light/dark cycle at room temperature and fed *ad libitum* with standard rodent diet. The endpoint of the study was set when the physical condition for more than 50% of remaining tumor-bearing mice was evaluated as critical and euthanized within 24 hours.

### Tumor Inoculation

Female wild-type mice and MCK-PGC-1α transgenic littermates (n = 8/group) at 4 month of age were injected subcutaneously with 10^6^ Lewis lung carcinoma cells in 100 µL sterile vehicle, phosphate-buffered saline (PBS), between the shoulder blades. Controls were injected in the same manner and location with PBS (n = 7 for wild-type mice, n = 5 for MCK-PGC-1α mice). The weights of the animals were recorded and their general health was monitored daily after tumor inoculation. Individuals deemed in poor condition unable to survive until the endpoint of study were euthanized and excluded from further analysis.

### Spectrophotometer Assays

OXPHOS assays were performed as previously described [28]. In brief, homogenates from quadriceps and gastrocnemius were prepared using a tissue homogenizer (Omni) in PBS plus protease inhibitor cocktail (Roche) on ice. Samples were centrifuged at 800 g for 5 minutes and the supernatant of the homogenate was added to a buffer containing 10 mM KH_2_PO4, 1 mg/mL BSA, 120 mM lauryl maltoside, and 2 mM cytochrome *c* reduced with sodium hydrosulfite in. The mixtures were followed at 550 nm with the absorption reading taken every 11 seconds for 2 minutes at 37°C. 240 µM potassium cyanide was used to inhibit the reaction to ensure slope was specific to cytochrome *c* oxidase (COX). The slopes were normalized by protein concentration determined by Bradford assay.

For citrate synthase (CS) activity assay, the supernatants were added to a buffer containing 50 mM Tris-HCl pH 7.5, 20 mM acetyl CoA, 10 mM 5, 5′-dithiobis-(2-nitrobenzoic acid), and 0.1% triton X-100. The assay was performed at 30°C with 50 mM oxaloacetate (OXA) to start the reaction. Readings were obtained every 11 seconds for 3 minutes. The slopes taken before adding OXA were extracted from the slope with OXA. Normalization was as above.

### mRNA Isolation and Reverse Transcriptase PCR

Dissected quadriceps and gastrocnemius muscle tissues were submerged in TRIzol® (Sigma/Invitrogen). Tissues were homogenized with a hand-held rotor homogenizer (VWR), and RNA was extracted by chloroform phase separation. We used 1 µg of RNA for reverse-transcription reaction using the iScript cDNA synthesis kit according to the manufacturer's protocol (BioRad).

### Real-time PCR

Maxima SYBR Green/ROX qPCR master mix (Fermentas) was used according to manufacturer's directions to perform real-time PCR. Primers used for the cDNA quantification were: *Ppargc1a* (5′- CTGCGGGATGATGGAGACA, 5′- AGCAGCGAAAGCGTCACA), *Ppargc1b* (5′- TGGCCCAGATACACTGACTATG, 5′- TGGGCCTCTTTCAGTAAGCT), *Ppara* (5′- TTCCCTGTTTGTGGCTGCTAT, 5′- CCCTCCTGCAACTTCTCAATGTAG), *Pparg* (5′- CGGAAGCCCTTTGGTGACTTTA, 5′- GCGGTCTCCACTGAGAATAATGAC), *Ppard* (5′- ACCGCAACAAGTGTCAGTAC, 5′- CTCCGGCATCCGTCCAAAG), and *Gapdh* (5′- TGCACCACCAACTGCTTAG, 5′- GGATGCAGGGATGATGTTC).

The following primer pairs were used for the quantification of mtDNA copy number in total DNA (extracted with phenol: chloroform phase separation): *Nd1* (5′- CAGCCTGACCCATAGCCATA, 5′- ATTCTCCTTCTGTCAGGTCGAA), *Actb* (5′- TCACCCACACTGTGCCCATCTACGA, 5′- CAGCGGAACCGCTCATTGCCAATGG). Comparative Ct method was used to determine the relative abundance of genes of interest or mtDNA [29].

### Western Blotting Analysis

Protein extracts were prepared from the quadriceps and gastrocnemius muscles that were homogenized with a hand-held rotor (VWR) in PBS containing protease inhibitor cocktail (Roche). Samples were then snap frozen in liquid nitrogen and stored in −80°C until used. Upon use, homogenates were diluted 1∶10 with RIPA buffer (62.5 mM Tris-HCl pH 7.4, 150 mM NaCl, 1% NP-40, 0.25% SDS, 1 mM EDTA, with protease inhibitors and phosphatase inhibitors added freshly) and sonicated briefly. Homogenates were then centrifuged at 15,000× g and the supernatant was collected. Proteins were quantified using Bradford assay. Equal amount of protein were loaded onto a 4–20% SDS-polyacrylamide gradient gel (BioRad). The gel was blotted on Polyvinylidene Fluoride (PVDF) membrane (BioRad).

Membranes were blocked in Odyssey blocking solution (LI-COR Biosciences) diluted 1∶1 with PBS for 1 hour at room temperature. Primary antibodies used were OXPHOS rodent cocktail (Mitosciences), α-tubulin (Sigma), PGC1-α (Santa Cruz), SDHA (Mitosciences), β-actin (Sigma), cytochrome *c* (Mitosciences), porin (Mitosciences), and UQCRC1 (Mitosciences). Primary antibody was incubated overnight at 4°C. Secondary antibodies used were either infrared conjugated antibodies anti-rabbit-700 or anti-mouse-800 (Rockland) at manufacturer-suggested concentrations. Secondary antibodies were incubated for 1 hour at room temperature. Blots with infrared secondary antibodies were visualized with Odyssey Infrared Imaging System (LI-COR Biosciences). Optical density measurements were taken using the Gel-Pro Analyzer software.

### Treadmill

Endurance was evaluated using a six lane treadmill with motivation grid designed for rodents (Columbus Instruments). Animals were given one training day to adapt to the equipment and motivation grid. On the test day, mice were required to run at a speed of 8 m/min for 5 minutes and the number of falls onto the motivation grid was recorded for each mouse.

### Serum IL-6 Quantifications

Blood was taken from the left ventricle of deeply anesthetized mice before euthanized. Blood was allowed to clot on ice, and serum was isolated at 1,000× g in a bench top centrifuge (Eppendorf 5424) for 15 minutes at 4°C. An additional centrifugation step of the serum at 10,000× g for 10 minutes at 4°C was performed for complete platelet removal. Serum was used in BD cytometric bead array mouse inflammation cytokine kit according to the manufacturer's instructions (BD Biosciences). Samples were analyzed on a BD LSRFortessa cell analyzer (BD Biosciences).

### Statistical Analysis

All results were expressed as means ± STDEV. Significance of the differences was evaluated by 2-way ANOVA followed by Bonferroni post-test for experiments with more than 2 groups or by unpaired Student t-test between 2 groups. Differences were considered significant when p<0.05 (*), 0.001<p<0.01 (**), p<0.001(***).
